# Data-driven gated PET/CT: implications for lesion segmentation and quantitation

**DOI:** 10.1186/s40658-021-00411-5

**Published:** 2021-08-28

**Authors:** M. Allan Thomas, Tinsu Pan

**Affiliations:** Department of Imaging Physics, UT MD Anderson Cancer Center, Houston, TX 77030 USA

**Keywords:** Data-driven gating, SUV, Misregistration, PET/CT

## Abstract

**Background:**

Data-driven gating (DDG) can improve PET quantitation and alleviate many issues with patient motion. However, misregistration between DDG-PET and CT may occur due to the distinct temporal resolutions of PET and CT and can be mitigated by DDG-CT. Here, the effects of misregistration and respiratory motion on PET quantitation and lesion segmentation were assessed with a new DDG-PET/CT method.

**Methods:**

A low-dose cine-CT was acquired in misregistered regions to enable both average CT (ACT) and DDG-CT. The following were compared: (1) baseline PET/CT, (2) PET/ACT (attenuation correction, AC = ACT), (3) DDG-PET (AC = helical CT), and (4) DDG-PET/CT (AC = DDG-CT). For DDG-PET, end-expiration (EE) data were derived from 50% of the total PET data at 30% from end-inspiration. For DDG-CT, EE phase CT data were extracted from cine-CT data by lung Hounsfield unit (HU) value and body contour. A total of 91 lesions from 16 consecutive patients were assessed for changes in standard uptake value (SUV), lesion glycolysis (LG), lesion volume, centroid-to-centroid distance (CCD), and DICE coefficients.

**Results:**

Relative to baseline PET/CT, median changes in SUV_max_ ± σ for all 91 lesions were 20 ± 43%, 26 ± 23%, and 66 ± 66%, respectively, for PET/ACT, DDG-PET, and DDG-PET/CT. Median changes in lesion volume were 0 ± 58%, − 36 ± 26%, and − 26 ± 40%. LG for individual lesions increased for PET/ACT and decreased for DDG-PET, but was not different for DDG-PET/CT. Changes in mean HU from baseline PET/CT were dramatic for most lesions in both PET/ACT and DDG-PET/CT, especially for lesions with mean HU < 0 at baseline. CCD and DICE were both affected more by motion correction with DDG-PET than improved registration with ACT or DDG-CT.

**Conclusion:**

As misregistration becomes more prominent, the impact of motion correction with DDG-PET is diminished. The potential benefits of DDG-PET toward accurate lesion segmentation and quantitation could only be fully realized when combined with DDG-CT. These results impress upon the necessity of ensuring both misregistration and motion correction are accounted for together to optimize the clinical utility of PET/CT.

**Supplementary Information:**

The online version contains supplementary material available at 10.1186/s40658-021-00411-5.

## Background

Respiratory motion affects positron emission tomography (PET) and computed tomography (CT) in unique ways when they are combined in a PET/CT scan. Modern PET acquisition times lead to a respiratory motion averaged PET scan—motion in the region of interest causes a smearing of the acquired counts across its range, leading to blurred images. The significantly faster CT acquisition leads to the possibility that a pronounced misalignment with the more temporally averaged PET data can occur if motion is significant. Clinically relevant misregistration between PET and CT can then appear, leading to PET images with poor diagnostic quality and inaccurate quantitation [1–4].

Much work has been pursued previously to address these issues, namely respiratory gating of both PET and CT [5–8]. Until very recently, the only method used for gating PET or CT in clinical scans was external device-based gating (EDG) [9]. When EDG is successful, a respiratory waveform can be deduced and the full cycle of respiratory motion and related phases can be established [7]. The need for an external device has many drawbacks [10]; however, so EDG for PET/CT has historically been associated with radiation therapy planning and rarely diagnostic PET/CT [9]. EDG requires a gating device, which must be available and integrated with the PET/CT scanner. It is also difficult to utilize EDG fully in the clinic because it adds time and effort to the clinical workflow—setting up the device takes both additional time and personnel training. Finally, for the most appropriate use of EDG, it should be included for both PET and CT in PET/CT studies so that the gated PET data can be matched with gated CT. This produces many of the same difficulties as already outlined above for EDG-PET.

Recent studies have shown that data-driven gating (DDG) methods are at least equal to [11, 12], if not better than [13], EDG. In DDG methods, the raw imaging data are processed to extract a motion signal directly from the anatomy experiencing the motion [11–18]. DDG methods for both PET and CT are certainly not new, but only recently has DDG-PET started to make its way into clinical practice with commercially available products. The current GE product utilizes principal component analysis [19, 20], while United Imaging calculates center of mass [21]. As DDG-PET continues to gain in clinical use, EDG-based methods for CT will become impractical to match with DDG-PET, requiring equally capable techniques for DDG-CT [10, 16, 22–24]. Some other recent work has combined prospective DDG-PET with end-expiration (EE) breath-hold CT to progress toward better matching between DDG-PET and CT [25, 26].

An important theme discussed in prior studies of DDG-PET is the acknowledgement that misregistration between DDG-PET and helical CT may have been present in some fraction of cases studied [11, 15]. Meier et al. showed previously that different CT phases being used for attenuation correction can lead to significant changes in PET quantitation, with the impact even more severe for DDG-PET than static PET [27]. However, to our knowledge, no previous work attempted to correct DDG-PET and CT misregistration before analyzing the impact of DDG-PET for the purposes of their study. For relevant anatomical sites in the upper abdomen and lower thorax, the effects of misregistration can become critical. It is possible that much of the previous work on DDG-PET was limited in its portrayal of the benefits of DDG due to the unavoidable inclusion of misregistered data in the analysis.

We recently implemented a new method that combines DDG-CT with GE’s DDG-PET to overcome the problems of PET/CT misregistration and inaccurate PET quantitation [10]. This new DDG-PET/CT involves a secondary cine-CT in the region of misregistration when it is identified before the end of a PET/CT acquisition and has been implemented on a network of seven PET/CT scanners. From the cine-CT data, both an average CT (ACT) and DDG-CT are extracted to provide improved registration with baseline PET and DDG-PET, respectively. This reduced scan coverage, cine-CT approach is an alternative to a limited scan range, repeat PET/CT when clinically relevant misregistration is identified during a PET/CT scan. It offers a lower radiation dose and a shorter scan time than repeat PET/CT [10] with the current vendor’s application because minimizing the repeat PET/CT to less than one PET bed is not practically achievable. The resulting DDG-PET/CT also led to improved registration and enhanced PET quantitation through increased standard uptake values (SUV).

In this work, our objective is to explore the separate effects of misregistration and motion correction on lesion segmentation and quantitation in PET/CT. We aim to understand the significance of these effects and how they may impact the clinical utility of PET/CT, both for general diagnostic and radiation therapy applications. We investigate these aims by comparing four PET/CT methods, with different combinations of PET and CT that may or may not involve DDG. Our results provide strong evidence that multiple metrics tied to the clinical assessment and treatment of cancer can change significantly depending on the use of DDG for PET and CT. Lesion SUV, volume, location, and glycolysis (LG) are all impacted by misregistration and respiratory motion, as well as whether or not these issues are corrected with DDG. Combining DDG-PET with DDG-CT offers the optimal registration between PET and CT and is the only way to ensure that the full benefits of DDG for PET are realized.

## Methods

### Patient selection

The study was approved by an Institutional Review Board. The need for informed written consent was waived. Sixteen consecutive patients who received the cine-CT protocol [10] due to PET/CT misregistration were collected, including 9 ^18^F-fluorodeoxyglucose, 6 ^68^Ga-DOTATATE, and 1 ^18^F-fluciclovine studies. Of the 16 cases studied, 12, 3, 1, and 1 involved liver, lung, bone, and lymph node lesions, respectively.

### PET/CT, average CT, and DDG-CT protocols

The scans were acquired using four different GE Discovery PET/CT scanners: D690, D710, DR, and DMI. The PET images were reconstructed with time-of-flight ordered subsets expectation maximization using 18 subsets and 2 iterations, including resolution recovery and a 5 mm Gaussian post-filter. The same helical CT protocol was used for all CT scanners: 120 kVp, pitch factor = 0.984, gantry rotation time = 0.5 s, X-ray collimation = 64 × 0.625 mm, noise index = 30, maximum mA = 560, minimum mA = 60 (without), and minimum mA = 100 (with) iodinated contrast injection. The cine-CT scan protocol was 120 kVp, 5 s cine scan duration, gantry rotation time = 0.8 s, X-ray collimation = 8 × 2.5 mm, noise index = 70, minimum mA = 10, and maximum mA = 20. Each segment of the cine-CT covered 2 cm and was for a 5 s duration, chosen to cover 97.5% of the normal respiration rates of patients > 65 years old [28]. Whether to initiate a cine-CT to correct for PET/CT misregistration was determined at the time of the scan by the imaging technologist. If the technologist identified any region of clinically relevant misregistration, not necessarily only a specific misregistered lesion, a cine-CT scan was initiated. Whatever scan range was deemed necessary to cover the region of misregistration was prescribed. There was no requirement for cine-CT coverage to be as large as a full PET bed. Cine-CT scan coverage ranged from 8 to 20 cm in this patient group.

DDG-PET was applied retrospectively to all PET beds where the respiratory motion threshold (*R* value) was at least 15 [20, 29]. The PET scan time was not increased for DDG-PET, the implications of which will be addressed later in this work. The cine-CT data were used to derive both ACT and DDG-CT, which were embedded into the helical CT in the cine-CT scan range, and then used for attenuation correction (AC) of the whole-body PET data [30, 31] and the DDG-PET data, respectively. The derivation of DDG-PET has been outlined previously [20]. The details related to EE CT derivation, as well as PET, helical CT, and cine-CT acquisitions, can be found in previous work [10].

### Quantitative measurements

Only lesions that were within the cine-CT scan range were included, yielding 91 with 82, 6, 2, and 1 from the liver, lung, bone (spine at level of upper abdomen), and lymph nodes (abdomen), respectively. Four different PET/CT methods were analyzed: (1) whole-body, or baseline, PET/CT, (2) PET/ACT, (3) DDG-PET, and (4) DDG-PET/CT. The different PET and CT datasets used, as well as their respective respiratory phase identifiers, are explained in Table 1. The lesions were contoured with three different techniques, including a threshold method based on maximum SUV (SUV_max_), a gradient-based auto-contouring method (PET Edge, MIM Software Inc.), and hand-drawn contours. In the threshold method, the nominal threshold was 40%, but for some lesions it was adjusted upwards until differentiation from background was achieved. Once the threshold percentage was established for a particular lesion, it was used for all four PET/CT methods. Our interests in this work were not related to identifying optimal contouring methods nor making distinct comparisons between contouring methods. We simply aimed to use a consistent contouring method so that quantitative measurements of lesions could be compared fairly based on PET/CT techniques. While we did observe some differences between contours based on the segmentation technique, nearly all of the relevant findings discussed later were consistent regardless of contouring method. Specific instances where contouring methods were important are identified when relevant.

**Table 1:** Summary of PET/CT methods used in this study

PET/CT method	PET data	Attenuation correction	PET respiratory phase	CT respiratory phase
Baseline PET/CT	Baseline PET	Helical CT	Average	Random
PET/ACT	Baseline PET	Average CT	Average	Average
DDG-PET	DDG-PET	Helical CT	End-expiration	Random
DDG-PET/CT	DDG-PET	DDG-CT	End-expiration	End-expiration

Lesion glycolysis (LG) can be used in addition to other quantitation metrics like SUV_max_ to assess patient status and lesion response [32]. Both individual LG and total patient LG (TLG) values were calculated and compared here, defined as follows:

$$\begin{aligned} LG & = Lesion\;SUV_{mean} \times Lesion\;Volume \\ TLG & = Total\;Lesion\;Volume\;SUV_{mean} \times Total\;Lesion\;Volume. \\ \end{aligned}$$

Centroid-to-centroid distances (CCD) and Dice similarity coefficients (DICE) were also calculated for the three modified PET/CT methods relative to the reference baseline PET/CT. DICE is a measure of overlap between volumes of interest [33]. Finally, lesion mean CT Hounsfield unit (HU) values were compared among the four PET/CT methods. The various metrics used for analysis are summarized in Table 2. Note that lesions were contoured only on the PET images. All metrics analyzed are related to PET data except for lesion mean HU, which was determined with the PET-based contour but used the CT image for determination of the HU value.

**Table 2:** Statistical summary of metrics used in this study

	PET/ACT			DDG-PET			DDG-PET/CT		
	$\tilde{x }$± σ	Range	*p*	$\tilde{x }$± σ	Range	*p*	$\tilde{x }$± σ	Range	*p*
*Metric ratio*									
Volume	1.00 ± 0.58	0.38–3.38	0.54	0.64 ± 0.26	0.20–1.61	***	0.74 ± 0.40	0.37–2.39	***
SUV_max_	1.20 ± 0.43	0.90–3.30	***	1.26 ± 0.23	0.58–1.81	***	1.66 ± 0.66	1.07–4.48	***
SUV_mean_	1.17 ± 0.40	0.93–3.28	***	1.19 ± 0.18	0.53–1.81	***	1.55 ± 0.52	1.05–3.50	***
LG	1.26 ± 1.23	0.46–6.21	*	0.78 ± 0.32	0.11–2.17	***	1.13 ± 1.12	0.46–7.23	0.19
TLG	1.12 ± 0.32	0.80–2.04	0.41	0.82 ± 0.15	0.54–1.13	**	1.10 ± 0.38	0.79–2.08	0.34
*Metric*^†^									
CCD	1.6 ± 2.5	0.2–15.1	‒	3.9 ± 3.6‡	0.3–18.2	***	4.6 ± 3.8‡	0.4–21.0	***
DICE	0.80 ± 0.15	0.26–0.94	‒	0.67 ± 0.18‡	0–0.94	***	0.64 ± 0.17‡	0–0.89	***
Lesion ΔHU	61.6 ± 311	− 65–906	***	− 8.7 ± 157	− 859–49	*	85.6 ± 352	− 64–953	***

$\tilde{x }$ = median, σ = standard deviation, LG = lesion glycolysis, TLG = total lesion glycolysis, CCD = centroid-to-centroid distance (mm), DICE = DICE similarity coefficient, ΔHU = change in mean HU

All metric ratios and tests of significance (*p*) are relative to baseline PET/CT except for ^‡^*p* relative to PET/ACT

^†^CCD, DICE, and ΔHU values are calculated relative to baseline PET/CT lesion contours

*p < 0.01, **p < 0.001, ***p < 0.0001

### Statistical analysis

A large majority of our datasets were non-normally distributed, so nonparametric significance tests were deemed more appropriate and median values were used for comparisons in most cases. For all matched group comparisons (repeated measures), Friedman’s test was used and then multiple comparisons were assessed for more specific analysis among groups. A false discovery rate correction using the two-state step-up method of Benjamini et al. [34] was also implemented using a false discovery rate of 0.01, with all *p* values reported after adjustment for multiplicity. For non-matched comparisons among more selective groups of data, Mann–Whitney tests were used. All statistical analyses were performed with GraphPad Prism 8.0.0 (GraphPad Software), and statistical significance was considered true for *p* < 0.01.

## Results

**Fig. 1:**
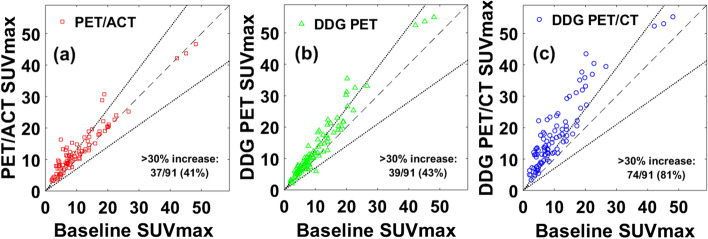
Scatter plots of SUV_max_ values compared to baseline PET/CT for **a** PET/ACT, **b** DDG-PET, and **c** DDG-PET/CT. The dotted line is the line of equivalence, and lines representing ± 30% change in SUV_max_ are also plotted in each figure. The number (%) of lesions with > 30% increase in SUV_max_ is included in each figure

Table 2 provides summary statistics for all the metrics tracked. Improved AC with ACT did not change lesion volumes, but DDG led to decreased lesion volumes for both DDG-PET and DDG-PET/CT. This difference was consistent regardless of lesion contouring method. SUV_max_ increased relative to baseline for all PET/CT methods, but was increased most for DDG-PET/CT (*p* < 0.0001). There was no statistically significant difference between SUV_max_ for PET/ACT and DDG-PET (*p* = 0.36). Note that the absolute increases were not quite as substantial for SUV_mean_ compared to SUV_max_, but all levels of significance in differences in SUV_max_ among the PET/CT methods applied to SUV_mean_ as well. Figure 1 shows SUV_max_ values for the three modified PET/CT methods relative to baseline PET/CT. All three methods had more than 1/3 of lesions with > 30% increase in SUV_max_, but DDG-PET/CT had the most with ~ 4/5 and a median increase across all lesions of 66%.

Relative to baseline PET/CT, LG increased for PET/ACT, decreased for DDG-PET, but did not change for DDG-PET/CT. Patient TLG was also smaller for DDG-PET but was not different from baseline PET/CT for PET/ACT or DDG-PET/CT. Statistical significance measures were slightly dependent on contouring method. The most consistent observations were decreased patient TLG for DDG PET and no change for DDG PET/CT, both relative to baseline PET/CT. There were some lesions with large CCD values for PET/ACT relative to baseline PET/CT, but the median distance was less than 2 mm. Upon application of DDG, the median CCD increased to ~ 4 mm for DDG-PET and ~ 4.5 mm for DDG-PET/CT. This difference in CCD between DDG-PET and DDG-PET/CT was not statistically significant (*p* = 0.50). The DICE coefficient results followed similar trends. PET/ACT certainly had some outlier lesions where DICE relative to baseline PET/CT was very poor (< 0.5), but the majority of lesions had DICE > 0.75. When correcting for motion with DDG, the median DICE coefficient decreased to ~ 0.65 from 0.80 and there were even lesions with no overlap at all (DICE = 0) between baseline PET/CT and DDG-PET or DDG-PET/CT. DDG-PET and DDG-PET/CT had no difference in DICE (*p* = 0.37).

**Fig. 2:**
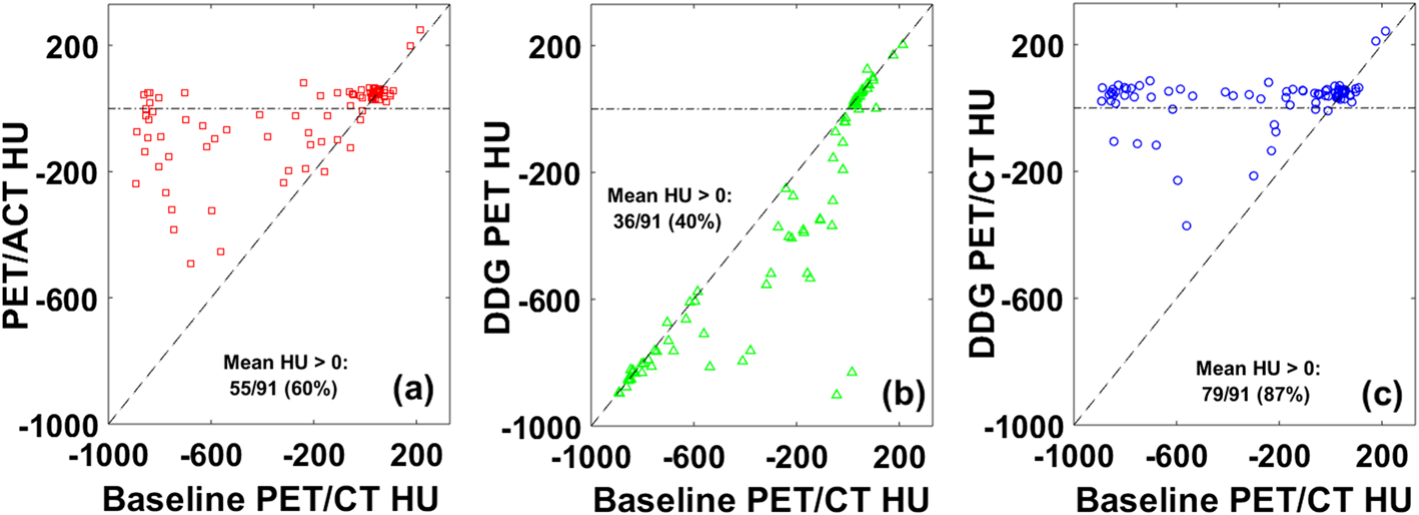
Scatter plots of lesion mean HU relative to baseline PET/CT for: **a** PET/ACT, **b** DDG-PET, and **c** DDG-PET/CT. In each figure, the number (%) of lesions with a mean HU > 0 is included. Note that at baseline, 38/91 (42%) lesions had mean HU > 0. The dashed line in each figure is the line of equivalence, while the dash-dot line is plotted at HU = 0

Figure 2 shows comparisons in lesion mean HU between the four PET/CT methods. PET/ACT generally led to significant increases in HU, especially for lesions with mean HU < 0 in baseline PET/CT. Such lesions were likely poorly registered initially (located in air) but then became more correctly registered in the ACT (located in soft tissue). On the other hand, DDG-PET tended to cause either little change in HU or relevant decreases in some lesions. This is due to changes in the location and/or volume of the lesion from gating with DDG, but the same helical CT is used for both baseline PET/CT and DDG PET/CT. As whole groups, the mean HU values were not different between PET/ACT and DDG-PET/CT (*p* = 0.10). But DDG-PET/CT further increased mean HU values, even beyond what was achieved with ACT, in many lesions. These results show that some lesions were still not ideally registered with ACT but DDG-CT could further improve registration and provide more accurate AC. Overall, improved registration with ACT increased the number of lesions with mean HU > 0 from 38 at baseline to 55 with PET/ACT, while DDG PET/CT further increased the number to 79. DDG PET decreased the number of lesions with mean HU > 0 to 36.

Table 3 presents SUV_max_ ratio and lesion mean ΔHU data for more specific groups of lesions based on distinctions in lesion volume and mean HU at baseline. The median lesion volume at baseline was ~ 3 cc so this threshold served to split the lesions into two equal sized groups. All modified PET/CT methods still showed significant increases in SUV_max_ relative to baseline PET/CT, regardless of the distinction in lesion volume. However, only for DDG PET/CT was there a clear difference in SUV_max_ ratios between small and large lesions. When looking at both small and large lesion groups, DDG-PET/CT had increased SUV_max_ relative to both PET/ACT and DDG-PET (all four: *p* < 0.0001). All advanced PET/CT methods also showed significant changes in lesion mean HU from baseline, even when separating lesions by volume. The only exception was DDG-PET for the small lesion group. Interestingly, there were significant differences in ΔHU between the small and large lesion groups, and this was true for all three modified PET/CT methods. For PET/ACT and DDG-PET/CT, small lesions maintained much larger increases in HU, while for DDG-PET large lesions had larger *decreases* in HU. Some final observations related to lesion size were that large lesions showed larger changes in location (larger CCD values) relative to baseline for DDG-PET (*p* = 0.002) and DDG-PET/CT (*p* = 0.0005) when compared to small lesions. However, DICE coefficients were not different between small and large lesions—most likely because small changes in location can still lead to poor overlap with small lesions, yet larger changes in location do not cause as much loss in overlap for large lesions.

**Table 3:** SUV_max_ ratio and ΔHU comparisons for various groups of lesions

	PET/ACT		DDG-PET		DDG-PET/CT	
	$\tilde{x }$± σ	*p*	$\tilde{x }$± σ	*p*	$\tilde{x }$± σ	*p*
*SUV*_*max*_* ratio*						
Volume < 3 cc	1.31 ± 0.52	***	1.31 ± 0.24	***	1.96 ± 0.76	***
Volume > 3 cc	1.13 ± 0.26	**	1.19 ± 0.21	***	1.64 ± 0.44	***
*Small versus large volume*^†^	‒	0.02	‒	0.02	‒	*
HU < 0	1.35 ± 0.48	***	1.27 ± 0.26	***	2.01 ± 0.69	***
HU > 0	1.04 ± 0.20	0.04	1.24 ± 0.19	***	1.44 ± 0.26	***
*HU* < *0 versus HU* > *0*^†^	‒	***	‒	0.33	‒	***
*Lesion ΔHU*						
Volume < 3 cc	322 ± 352	***	− 4.9 ± 139	0.17	562 ± 395	***
Volume > 3 cc	20.2 ± 182	*	− 25.2 ± 168	*	50.5 ± 206	**
*Small versus Large Volume*^*†*^	‒	**	‒	*	‒	**
HU < 0	322 ± 321	***	− 32.1 ± 161	*	461 ± 339	***
HU > 0	5.6 ± 25.7	0.02	-0.7 ± 138	0.53	4.4 ± 25.4	0.03
*HU* < *0 versus HU* > *0*^†^	‒	***	‒	***	‒	***

$\tilde{x }$ = median, σ = standard deviation, cc = cm^3^, ΔHU = change in mean HU

Tests of significance (*p*) are relative to baseline PET/CT except those labeled with ^†^

SUV_max_ ratio and ΔHU values are calculated relative to baseline PET/CT

baseline volume < 3 cc (*n* = 45), baseline volume > 3 cc (*n* = 46)

baseline HU < 0 (*n* = 53), baseline HU > 0 (*n* = 38)

*p < 0.01, **p < 0.001, ***p < 0.0001

In line with the results presented in Fig. 2, a threshold of 0 for lesion mean HU at baseline was used to create two lesion groups based on initial registration with the helical CT. As can be seen in Table 3, the group of lesions with baseline HU > 0 saw no statistically significant change in HU relative to baseline for any of the modified PET/CT methods. This result provides support for the idea that lesions with baseline HU > 0 could be considered well-registered even with the baseline helical CT. Furthermore, 52 of the 91 lesions were identified as clearly misregistered by visually comparing baseline PET/CT with DDG PET/CT. All but one of these 52 lesions had baseline mean HU < 0. Then 37 of the remaining 39 lesions that were not visually misregistered had baseline mean HU > 0. So the use of lesion mean HU = 0 as a threshold and surrogate for determining PET/CT registration has strong support.

PET/ACT SUV_max_ was not different from baseline PET/CT for lesions with HU > 0. This is not surprising considering the main way SUV can increase in PET/ACT is through a large change in registration. But all other modified PET/CT methods had clear increases in SUV_max_ over baseline regardless of baseline lesion HU. For PET/ACT and DDG-PET/CT, there were also strong differences in SUV_max_ when comparing lesion groups on the basis of HU at baseline. But in DDG-PET the two lesion groups were equivalent. Finally, as expected, lesions that were misregistered at baseline had much more dramatic increases in HU for both PET/ACT and DDG-PET/CT. But for DDG-PET, misregistered lesions had a significantly larger decrease in HU. DDG-PET/CT also had slightly larger increases in HU than DDG-PET for lesions with baseline HU > 0 (*p* = 0.006). This difference leads to a final key result for SUV_max_ comparisons among lesions with baseline HU > 0: DDG-PET/CT also showed increased SUV_max_ relative to DDG-PET (*p* = 0.001).

**Fig. 3:**
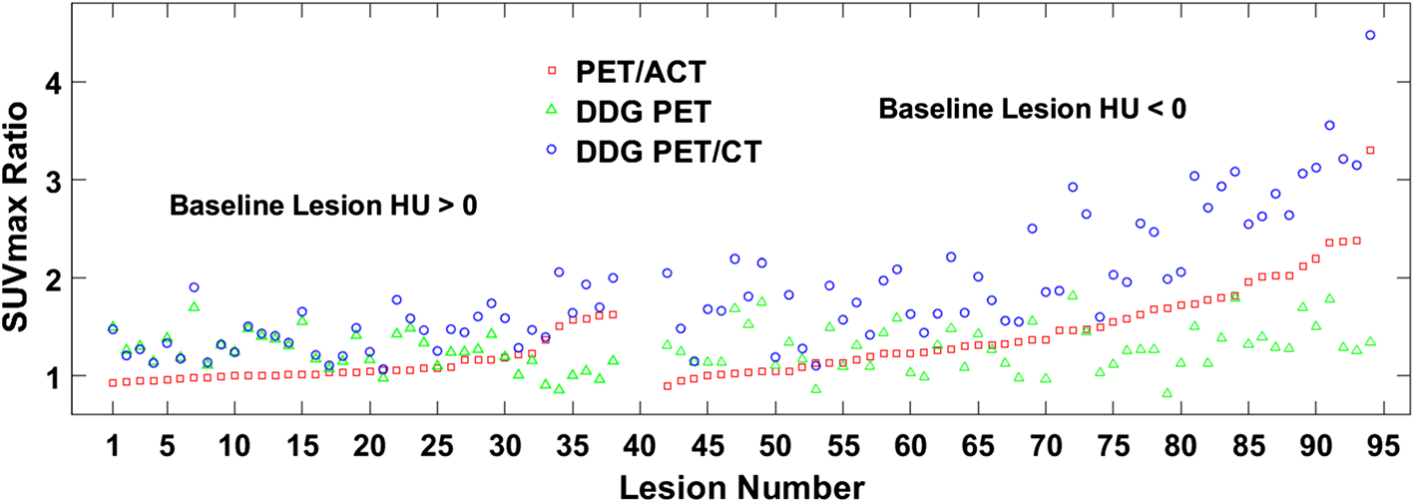
SUV_max_ ratios relative to baseline PET/CT. The ratios for each PET/CT method are plotted in increasing order based on the value for PET/ACT, but separated into two groups with baseline lesion mean HU > 0 on the left (#1–38) and baseline lesion mean HU < 0 on the right (#42–94)

Figure 3 plots the SUV_max_ ratios for PET/ACT, DDG-PET, and DDG-PET/CT relative to baseline PET/CT. All lesions, regardless of baseline HU value, tended to show the same trend: as the SUV_max_ ratio for PET/ACT increases, there is a larger separation in SUV_max_ ratios between DDG-PET and DDG-PET/CT. This trend is due to the similar effects of improved registration with ACT and DDG-CT on increasing SUV_max_. For lesions with baseline HU < 0, the impact of DDG-PET/CT is very clear—there is a large difference in SUV_max_ ratios between DDG-PET and DDG-PET/CT. Additionally, for many lesions with baseline HU < 0, the increase in SUV_max_ from improved PET/ACT registration was greater than that from PET motion correction with DDG-PET. But even with lesions having baseline HU > 0, there was a visible effect from DDG-CT in nearly half of them—the increased SUV_max_ ratio for DDG-PET/CT relative to DDG-PET was nontrivial. For lesions with poor registration in baseline PET/CT, the benefits of improved quantitation from PET motion correction were not realized without appropriate registration using DDG-CT. And even for lesions that were not clearly misregistered at baseline, the full impact of DDG for PET was not always achieved unless matched with DDG-CT.

**Fig. 4:**
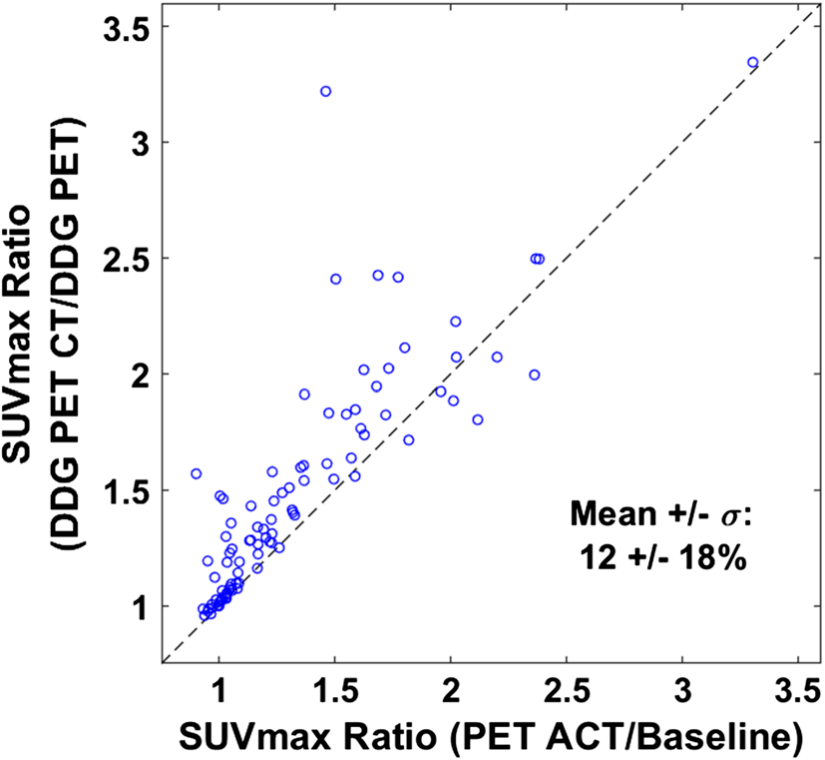
Scatter plot of SUV_max_ ratios: DDG-PET/CT relative to DDG-PET vs PET/ACT relative to baseline PET/CT. The dashed line is the line of equivalence. The mean and standard deviation of the % increase in the DDG-PET/CT relative to DDG-PET ratio are included

Figure 4 compares SUV_max_ ratios from different PET/CT methods, with the objective being to isolate the effect of DDG-CT compared to ACT. The SUV_max_ ratio of DDG-PET/CT to DDG-PET is compared to the ratio for PET/ACT to baseline. DDG-PET and DDG-PET/CT should maintain the same effect on SUV_max_ from the gating process, but the impact of DDG-CT on improved registration only becomes visible with DDG-PET/CT. As seen in Fig. 4, when comparing these ratios, DDG-CT improved SUV quantitation more than ACT in general. The mean improvement was 12%, and 80/91 (88%) lesions had a higher ratio from DDG-PET/CT relative to DDG-PET.

## Discussion

This study explored the effects of improved registration (ACT, DDG-CT) and motion correction (DDG-PET) on lesion quantitation and segmentation. Overall, our results show that SUV increased for all modified PET/CT methods relative to baseline PET/CT. But the most significant change in terms of absolute increase and the number of lesions with a clinically relevant (> 30%) increase was for DDG-PET/CT. To our knowledge, the increases in SUV_max_ observed here for DDG-PET/CT were larger than any other study of previous DDG-PET or DDG-PET/CT techniques. Some prior DDG-PET studies have typically noted an approximate increase in SUV_max_ of 10% from the DDG process [11, 25, 35], whereas in another study the DDG-PET method most closely resembling ours used here led to an approximate increase of 20% [15]. A very recent study that used the same DDG-PET method as here, but with an EE breath-hold CT protocol, found very similar increases in SUV_max_ as observed here for DDG-PET (~ 25%) [26].

SUV_max_ is the most common metric tracked clinically to assess lesion avidity and treatment response [32]. Since DDG uses only ~ 50% of PET data, there may be concern that the SUV_max_ increases are due to noise rather than a fundamental increase in SUV. But SUV_mean_ increased for DDG-PET and DDG-PET/CT as well, and all levels of statistical significance in differences between the various PET/CT methods were the same whether SUV_max_ or SUV_mean_ were compared. While the added noise may cause a consistent increase to SUV_max_, it should lead to a more random effect on SUV_mean_. This supports the idea that the consistent, statistically significant increases in SUV_max_ and SUV_mean_ observed in our data for DDG-PET and DDG-PET/CT are not random nor explained by the increased noise from fewer PET counts. Overall, the results presented here indicate that any increase in SUV_max_ from noisier DDG-PET data is only a small, insignificant portion. The same result has been suggested and shown previously in other work on DDG-PET, where analyses of noise and changes in SUV_max_ for DDG-PET and reduced-count non-gated PET were presented [11, 15, 18].

Lesion volumes were not affected by changes in registration, but the gating process from DDG reduces volume. The typical amount of volume decrease observed in this study was larger than, but still similar to, previous work on DDG-PET [11, 25, 26, 35]. The larger changes in volume observed in this work may result from this study’s selection bias—only prospectively identified cases with clinically relevant misregistration. The changes in SUV and lesion volume also affected LG and TLG. Because SUV increased but lesion volume remained unchanged from baseline to PET/ACT, LG also increased. But the significant decrease in lesion volume for DDG-PET overwhelmed the increase in SUV and LG also decreases. On the other hand, DDG-PET/CT saw no change in LG due to a further increase in SUV that offset the volume decrease from DDG. These distinctions in LG are important to identify and understand when such metrics are used to assess treatment response or other aspects of patient care. Both poor registration and the effects of motion can make LG values appear to be inaccurate.

The results in Fig. 2 show that the primary factor leading to increases in SUV_max_ for PET/ACT is increases in lesion mean HU, but DDG-PET either caused very little change or a significant decrease in mean HU. This suggests that PET motion correction from DDG does not lead to improved registration and more accurate AC, but rather the gating process from DDG is the main contributor to increased SUV_max_ for DDG-PET. DDG-PET/CT had the strongest impact on changes in HU, increasing the number of lesions with mean HU > 0 to 79 from only 38 at baseline. With respect to the effects of lesion volume on changes in SUV_max_ and lesion HU, the overall results were perhaps not quite as expected. DDG PET/CT was the only method where SUV_max_ ratios differed significantly between small and large lesions, with small lesions showing a larger increase in SUV_max_. Additionally, for PET/ACT and DDG-PET/CT, small lesions maintained much larger increases in HU than large lesions. SUV_max_ increased more for lesions with baseline HU < 0 in PET/ACT and DDG-PET/CT, but in DDG-PET the two lesion groups were equivalent. Finally, in lesions with baseline HU > 0, DDG-PET/CT led to larger increases than DDG-PET for both mean HU *and* SUV_max_. This result indicates that even for lesions that likely have little room for improved registration—no statistically significant change in HU relative to baseline from the application of DDG-PET or DDG-PET/CT—clear differences in both ΔHU and SUV_max_ still manifested from the use of DDG-CT.

Figure 3 supports these ideas further. For lesions with baseline HU < 0, DDG-PET and DDG-PET/CT had quite different SUV_max_. While the benefits of PET motion correction for increasing SUV_max_ are clear in general, the increase is only relevant and optimized if the lesion is well-registered. Proper AC from ACT was able to increase SUV_max_ much more than gating from DDG for many of these poorly registered lesions. Figure 4 also shows that DDG-PET/CT increased SUV_max_ relative to DDG-PET more than PET/ACT did relative to baseline PET/CT. This observation is likely a combination of: (1) some lesions really were better registered with DDG-CT than with ACT, and (2) some lesions were *more* poorly registered with DDG-PET than at baseline, giving DDG-CT an opportunity for even more improvement than ACT. The change in lesion HU data for DDG-PET in Fig. 2b provides evidence for #2 above. Additional file 1: Fig. S1 shows two example cases, one each from the liver and lung, that further support these ideas. In each case it is clear that registration was poor with baseline PET/CT and then slightly worse with DDG-PET. PET/ACT improved registration significantly, but it was not as optimized as what was achieved with DDG-PET/CT.

One limitation of this study is the moderate number of patients combined with the mix of different radiotracers used. The results may be partially affected by specific radiotracers. Our goal here was to ensure this study used data from consecutive patients that had received the DDG PET/CT protocol, and therefore, we did not make any specific selections on the basis of radiotracer. Recent work has shown that the use of DDG-PET as applied in this study (GE’s Q.Static, using a motion threshold) can be affected by specific radiotracers [26]. The DDG-PET/CT approach implemented here is enabled by a prospective cine-CT that is initiated only when PET/CT misregistration is identified in the clinic. It is not a fully prospective DDG protocol as discussed or used in previous work [11, 13, 25, 26]. Another limitation is therefore the inherent selection bias for cases studied here. But as was mentioned in the Introduction, previous studies have indicated that misregistration between DDG-PET and helical CT was a potentially significant limitation of their own results [11, 15]. This is particularly relevant when trying to understand the tradeoffs between increased noise, longer scan times, and the many benefits of DDG-PET [10, 11, 14].

These issues may also help to partially explain the disparity among previous studies, as well as compared to the results in this study, regarding how much SUV_max_ increases with DDG-PET. For example, despite the use of an EE breath-hold CT protocol, Kang et al. saw no difference in DDG-PET quantitation when compared with free-breathing CT [25]. This group also showed relatively low median increases in SUV_max_ with DDG-PET. A different study that also used EE breath-hold CT for prospective DDG-PET saw much larger increases in SUV_max_ after gating [26]. Different methods for DDG-PET and the CT used for attenuation correction likely will lead to a range of results, but it is also possible that certain techniques such as breath-hold CT are less reliable and difficult to reproduce in all patient populations. As shown in this study, DDG-PET data that is poorly registered with helical CT does not tell the complete story of the advantages of DDG for PET/CT. It is important that options be developed to deal with these issues while also minimizing the impact on patient workflows so that the use of DDG-PET can grow clinically. Other strategies have been explored, including a method that combines raw data analysis of helical CT (chest velocity) with the waveform produced from DDG-PET to better match DDG-PET with DDG-CT [24].

For clinics with more limited daily patient throughput in their PET/CT scanners, prospective DDG may not strongly affect the overall clinical workflow or number of patients scanned in a day. But at busy centers like our institution, most PET/CT scanners see > 20 patients per day so the increased time associated with prospective DDG could impact the daily clinical workflow. At present, the cine-CT approach applied in this work remains a practical choice for an institution like ours, offering improvement to the accuracy and clinical utility of key metrics extracted from PET/CT images in cases with relevant misregistration. It is possible that relevant cases of misregistration are missed due to reliance on imaging technologists. Additionally, in its current embodiment, it is difficult to justify adding the cine-CT in a prospective manner for a wider range of patients when many cases may not have clinically relevant misregistration. Improvements to the DDG PET/CT protocol are therefore possible, such as automated determination of misregistration with artificial intelligence and a thorough assessment of the minimal radiation dose needed for the cine-CT. Both of these topics will be pursued in future work and could open up the use of the DDG PET/CT methods described here to more patients.

## Conclusion

The effects of misregistration and motion correction were explored in detail by analyzing four separate, but related, PET/CT methods. Improved registration over helical CT with ACT leads to SUV increases, but it does not yield significant changes in lesion segmentation. DDG-PET reduces lesion volumes and shifts lesion locations. It also causes relevant changes in LG and TLG unless DDG-CT is used to provide more accurate registration. DDG-PET and the CT used for AC must be well registered to facilitate the benefits of DDG—the baseline helical CT may not be sufficient. Lesions that are poorly registered with helical CT may not provide sufficient accuracy for clinically relevant information that can be critical for their diagnosis, treatment, and response assessment with PET/CT imaging. DDG-CT offered the best registration between PET and CT studied in this work, provided the optimal SUV, and did not misrepresent LG or TLG. These results showcase the potential for significant clinical impact when both misregistration and motion correction are accounted for together in PET/CT.

## Supplementary Information

**Additional file 1: FIg. S1.** (From left to right): Baseline PET/CT, PET/ACT, DDG-PET, and DDG-PET/CT of (A) a liver study and (B) a lung study. The top images are fusions while the bottom images are only PET. SUVmax values for the lesions highlighted are included in the PET images.

## Data Availability

Research data are stored in an institutional repository and will be shared upon request to the corresponding author.

## Abbreviations

AC: Attenuation correction
ACT: Average CT
CCD: Centroid-to-centroid distance
CT: Computed tomography
DDG: Data-driven gating
DICE: DICE similarity coefficient
EDG: External device gating
EE: End-expiration
HU: Hounsfield unit
LG: Lesion glycolysis
PET: Positron emission tomography
SUV: Standard uptake value
TLG: Total lesion glycolysis
