# Supplementary material for: Data-driven gated PET/CT: implications for lesion segmentation and quantitation
Source: EJNMMI Phys. 2021 Aug 28;8:64. doi: 10.1186/s40658-021-00411-5 (PMC8403089; doi:10.1186/s40658-021-00411-5)
Supplement: Supplementary file 1 — Additional file 1: FIg. S1. (From left to right): Baseline PET/CT, PET/ACT, DDG-PET, and DDG-PET/CT of (A) a liver study and (B) a lung study. The top images are fusions while the bottom images are only PET. SUVmax values for the lesions highlighted are included in the PET images. [file 40658_2021_411_MOESM1_ESM.pdf]

Supplemental Data

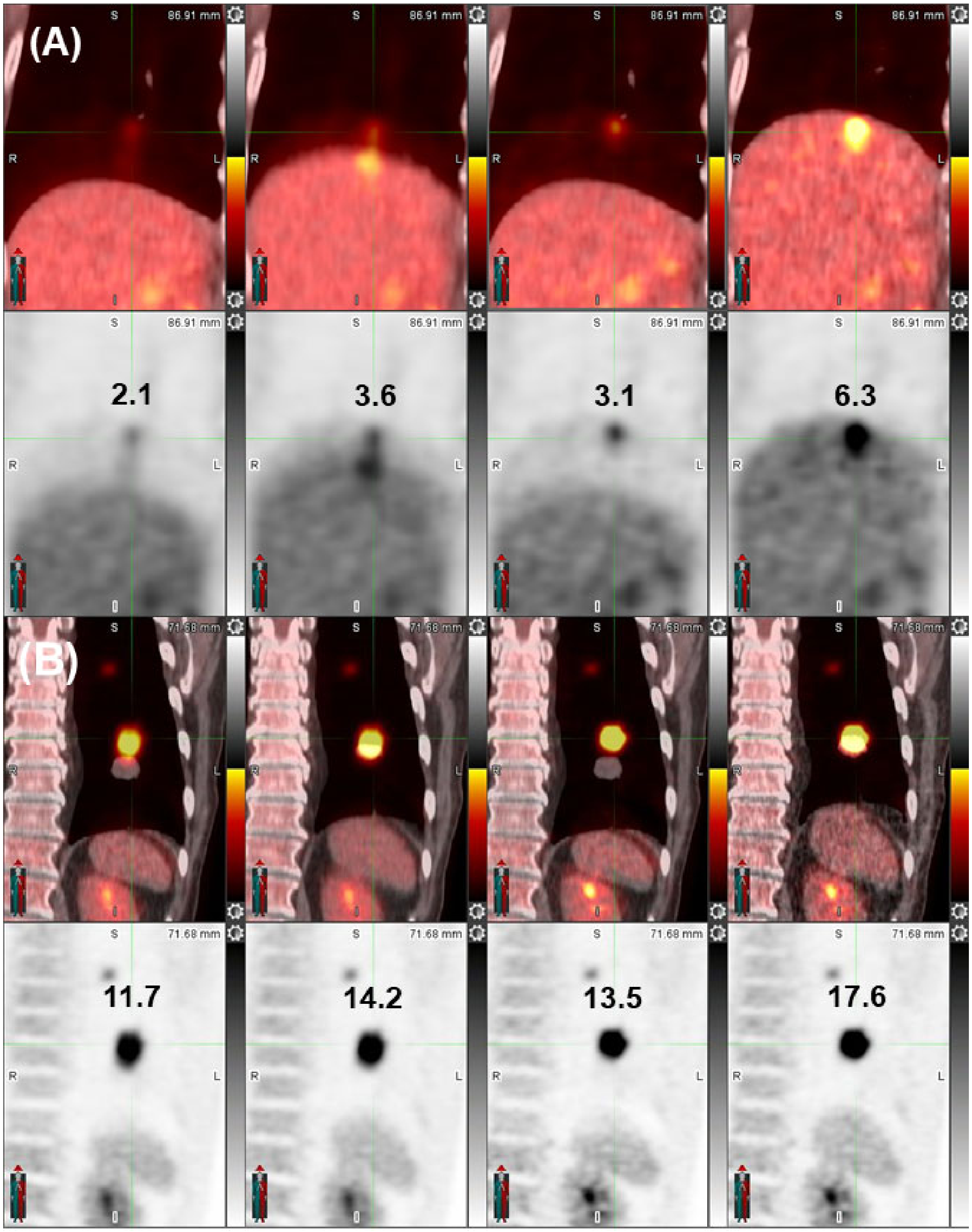

Figure S1. (From left to right): Baseline PET/CT, PET/ACT, DDG-PET, and DDG-PET/CT of (A) a liver study and (B) a lung study. The top images are fusions while the bottom images are only PET. SUV_{max} values for the lesions highlighted are included in the PET images.
